# Fast shoreline erosion induced by ship wakes in a coastal lagoon: Field evidence and remote sensing analysis

**DOI:** 10.1371/journal.pone.0187210

**Published:** 2017-10-31

**Authors:** Luca Zaggia, Giuliano Lorenzetti, Giorgia Manfé, Gian Marco Scarpa, Emanuela Molinaroli, Kevin Ellis Parnell, John Paul Rapaglia, Maria Gionta, Tarmo Soomere

**Affiliations:** 1 Istituto di Scienze Marine, Consiglio Nazionale delle Ricerche, Venezia, Italy; 2 Dipartimento di Scienze Ambientali, Informatica e Statistica, Università Ca’ Foscari, Venezia, Italy; 3 Centre for Tropical Environmental and Sustainability Sciences, James Cook University, Queensland, Australia; 4 Sacred Heart University Department of Biology, Fairfield, CT, United States of America; 5 Department of Cybernetics, School of Science, Tallinn University of Technology, Tallinn, Estonia; Universidade de Aveiro, PORTUGAL

## Abstract

An investigation based on *in-situ* surveys combined with remote sensing and GIS analysis revealed fast shoreline retreat on the side of a major waterway, the Malamocco Marghera Channel, in the Lagoon of Venice, Italy. Monthly and long-term regression rates caused by ship wakes in a reclaimed industrial area were considered. The short-term analysis, based on field surveys carried out between April 2014 and January 2015, revealed that the speed of shoreline regression was insignificantly dependent on the distance from the navigation channel, but was not constant through time. Periods of high water levels due to tidal forcing or storm surges, more common in the winter season, are characterized by faster regression rates. The retreat is a discontinuous process in time and space depending on the morpho-stratigraphy and the vegetation cover of the artificial deposits. A GIS analysis performed with the available imagery shows an average retreat of 3˗4 m/yr in the period between 1974 and 2015. Digitization of historical maps and bathymetric surveys made in April 2015 enabled the construction of two digital terrain models for both past and present situations. The two models have been used to calculate the total volume of sediment lost during the period 1968˗2015 (1.19×10^6^ m^3^). The results show that in the presence of heavy ship traffic, ship-channel interactions can dominate the morphodynamics of a waterway and its margins. The analysis enables a better understanding of how shallow-water systems react to the human activities in the post-industrial period. An adequate evaluation of the temporal and spatial variation of shoreline position is also crucial for the development of future scenarios and for the sustainable management port traffic worldwide.

## Introduction

Shoreline erosion and morphological changes due to storms, climate change and sea level rise have received a great deal of attention in both the scientific and popular literature over the past decade, particularly as the rates of these processes seem to be increasing [1–3]. Shoreline erosion has wide ranging detrimental impacts, from economic impacts due to property destruction and tourism reduction, and to ecological impacts resulting from the loss of sensitive habitats [4–6]. Erosion is a natural process of change over time, but often it is caused or amplified by humans through, e.g., subsidence induced by groundwater extraction [7–9], the construction of hard coastal protection structures [10], or the reduction of river sediment inputs into coastal systems [11,12]. All these processes have been extensively covered by scientific investigation. The impact of vessel navigation on coastal systems has largely been ignored, even though this process may be the most important factor controlling shoreline morphology in relatively sheltered coastal segments. Namely, in many coastal systems the background level of energy is low and ship wakes can become the major source of energy [13]. This is the case, for example, in enclosed basins such as estuaries, coastal lagoons, embayments, and inland or intra-coastal waterways [14–16]. The problem emerged when high-speed passenger ferries were introduced in the 1980s, and large and fast high-speed craft capable of carrying passengers and vehicles became common in the 1990s, with new and significant adverse effects being observed in numerous locations worldwide [14, 17–20]. In this context, little attention has been given to the dynamics of ship-channel interactions and their role in the local morphodynamics.

For vessels moving at finite depth in confined channels, depression wakes, or Bernoulli wakes, can become more important than other perturbations [21–23]. Depression wakes are normally generated by displacement type vessels, and their amplitude increases with an increase in the blocking coefficient (*S*, which is the ratio of the product of ship width and draught to the cross-sectional area of the channel), and ship velocity, usually expressed as the depth-based Froude number (*Fr*) [24]. These wakes can have a major role in sediment resuspension and redistribution [15, 25–28]. For a sailing regime characterized by relatively high *S* and *Fr* depression wakes of considerable amplitude can form when shoaling occurs at the channel sides. If navigation channels cross shallow lagoons, these wakes can propagate far into the adjacent shallow-water areas as highly non-linear asymmetric waves [23] with a steep rear face. Such entities often induce intense turbulence over a range of several hundreds of meters from the channel, with potentially high impact on the morphology of marginal areas [29].

In addition to morphological changes, vessel wakes can lead to the resuspension of contaminated sediments [25, 30–31]. In the long term, sediment resuspension can also affect the ecosystem by reducing water transparency [32] with negative effects on the bottom vegetation and phytoplankton abundance, trophic cascades in biological food webs [33], and disturbances on the development of juvenile fish communities [34–36]. Wake waves can also be hazardous to people along beaches and in coastal lagoons [14, 19, 37–38].

**Fig 1:**
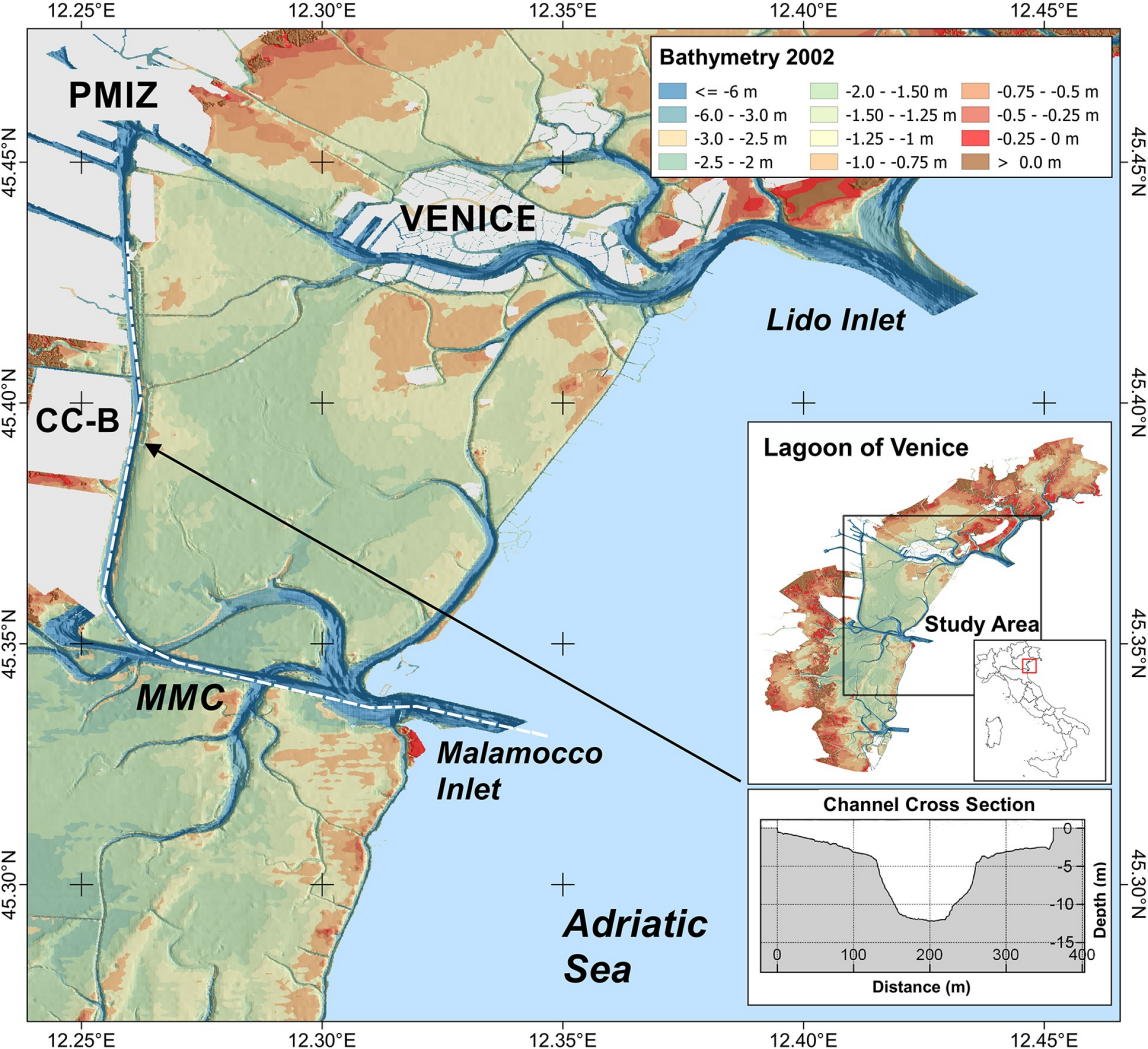
Map of the study area. The white dashed line marks the pattern of the Malamocco˗Marghera channel (MMC), the waterway connecting Malamocco inlet with the Porto Marghera Industrial Zone (PMIZ). The insets in the map show the location of the study area Cassa di Colmata B, CC-B) and the bathymetric profile of the navigation channel in the investigated sector. Data source: open database CIGNO [39].

In the Lagoon of Venice, Italy, depression wakes created by vessels in transit along the Malamocco Marghera Channel (MMC) (Fig 1) have a significant impact on the morphodynamics of the waterway and the nearest wetlands and mudflats.

This channel has been recently the subject of discussions as the local port authority is planning to create a new channel connecting the MMC to the passenger terminal in Venice, to redirect cruise ships to the MMC after the central government put a ban on the transit of vessels above 40,000 tons in the historic center of Venice. The proposal to reroute cruise ships from the Lido inlet to the MMC will avoid the historic center of Venice, but will increase the number of large ships in transit in the MMC with *S* almost twice that of cargo ships currently using the channel [40]. It is likely that implementation of this solution will result in higher impacts to the lagoon areas in the vicinity of the channel and rise maintenance costs.

During field surveys to investigate the characteristics of ship wakes and the associated sediment resuspension in the central lagoon, fast shoreline erosion was observed on the west side of the MMC. An investigation on the morphological evolution of the area was then undertaken combining on-site observations and measurements (short-term analysis) with remote sensing and integrating data in a GIS system (long-term analysis). This paper describes and discusses the results of our analysis of the temporal and spatial variation of the shoreline to estimate the total loss of sediment in the 40 years period from the channel construction (1974˗2015). The aim of the investigation is to evaluate the relative importance of erosion induced by ship traffic in the general context of morphological changes that occurred in the central Lagoon of Venice. Besides the specific problem of Venice and its port, understanding the effects of navigation in confined channels surrounded by shallow water areas [23, 29] may be of help in addressing technical solutions for similar situations worldwide, improving criteria for environmental decision making and sustainable management of ecosystem services.

## Study area

The study area is located on the western side of the MMC south of the Porto Marghera Industrial Zone (PMIZ) (Fig 1) in the Lagoon of Venice (Italy). The channel, built in the late 1960s, crosses mudflats and marshland areas of the central lagoon basin. It is an important internal waterway which permits direct access of commercial ships to the PMIZ from the Adriatic Sea, bypassing the historic center of Venice. The channel cross-section is trapezoidal and is larger in the E˗W reach where it is about 200 m wide and 17 m deep, while the N˗S reach narrows to 120 m with a depth of 12 m (Fig 1). Channel bottom width is 100 m and 60 m respectively in the two reaches. Sediments dredged during the channel construction were used to fill large marshland areas on the western bank which were reclaimed for further expansion of the PMIZ. Therefore the current stratigraphy of the reclaimed areas is characterized by lagoon sediments originally present in the area overlaid with material from the deep dredging of the channel.

The reclaimed areas, named *Casse di Colmata*, were initially designed to be at an elevation of 2 m above mean sea˗level. Adjacent to the channel they were contained within a bulkhead which was protected by rip˗rap. The project of creating a new industrial district was eventually abandoned and the reclaimed areas were left with no further disturbance until they were covered by dense vegetation, mostly shrubs in the north, with patches of poplar and birch trees in the center-south. At present, they are considered important biotopes for different bird species (Attachment 1 of European Council Directive 79/409/EEC of 2 April 1979 on the conservation of wild birds [41]).

According to the statistics of the port of Venice a total of 3400 vessel calls were recorded in 2015. About 500 of these were cruise calls directed to the passenger terminal via the inlet of Lido (Fig 1). Therefore approximately 2900 vessel (which include general cargo, dry and liquid bulk carrying vessels) calls to the PMIZ are through the MMC [42, 43] which means a total of at least 5800 vessel transits. Port traffic has increased over the last decades both in terms of size and the number of vessels. Currently the Port of Venice is accepting Post-Panamax and Post-Panamax Plus vessel types [44].

**Fig 2:**
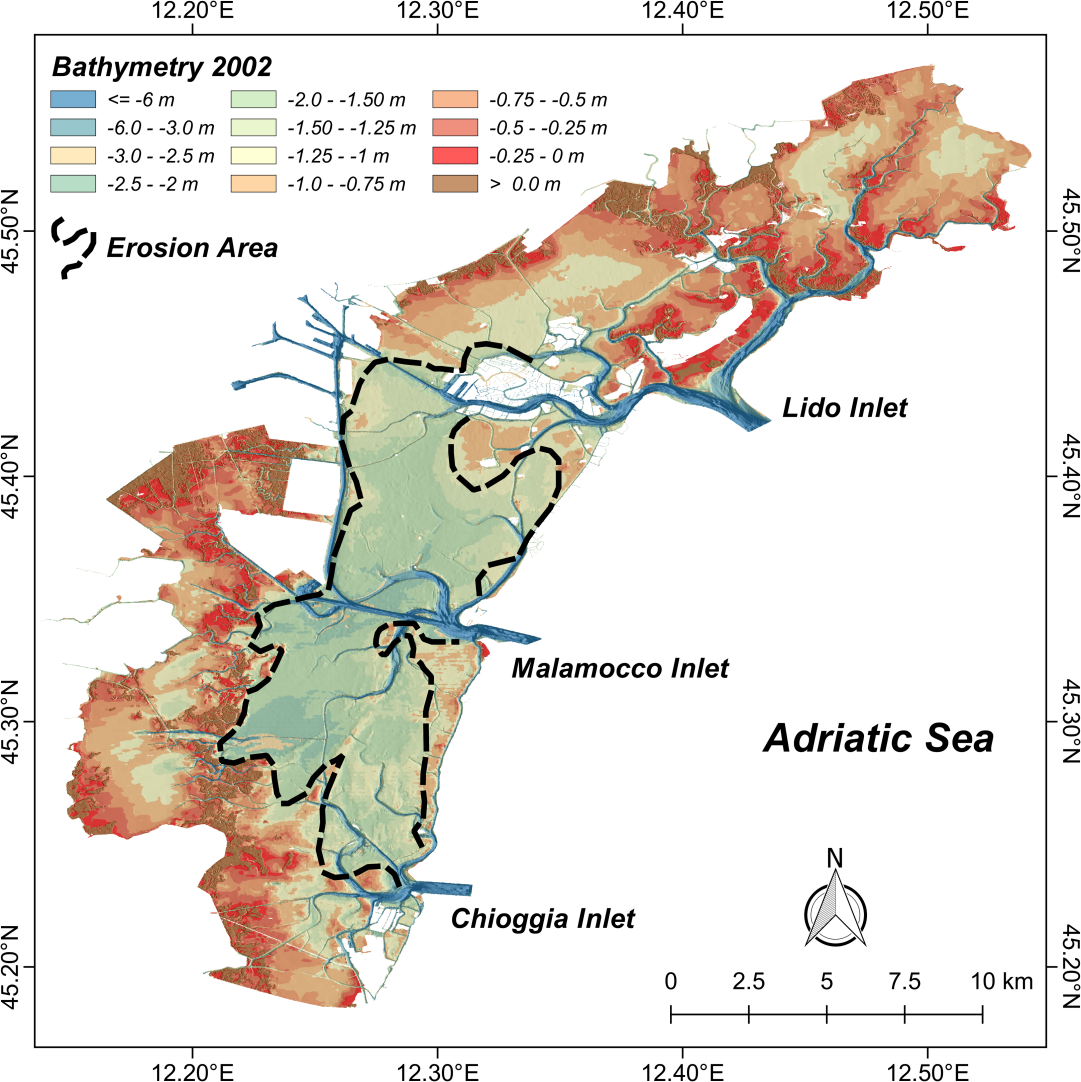
Bathymetric map of the Lagoon of Venice. Colour-shaded bathymetric map of the Lagoon of Venice. The dashed black line identifies the central lagoon area affected by significant erosion and flattening of the morphology, as described in [47]. Data source: open database CIGNO [39].

A number of studies have provided evidence of significant changes that have occurred to the morphological and hydrological conditions of the central area of the Lagoon of Venice after the opening of the MMC waterway, including erosion [45–47]. Molinaroli et al. [45] analyzed bathymetric changes occurred between 1970 and 2002. They found that 80% of the central lagoon area was affected by erosion, with a loss of more than 3 km^2^ of saltmarshes [45]. The area near the MMC was also characterized by widespread erosion of tidal flats with the movement of sediments resulting in deposition in the adjacent channels, leading to a general flattening of the morphology (Fig 2) [47].

Even though large ships in the Lagoon of Venice travel at low speeds and classical Kelvin wave effects are minimal, vessel-generated single depressions in this waterway can be as deep as 2.5 m [23]. These perturbations are highly nonlinear and propagate as long-living solitary waves of depression to a few hundreds of meters from the channel margin [29]. The largest depressions rapidly lose a large part of their height when breaking over the first 100-200 m of the mudflats creating resuspension of the bottom muddy sediments and erosion [25, 28]. The amplitude of the depression wave in the shallow lagoon only weakly depends on the initial amplitude of the wave, as demonstrated by Rodin et al. [29]. Therefore, even relatively modest depression waves, may penetrate to substantial distances into the lagoon provided their amplitude is large enough to ensure a strongly nonlinear regime of propagation. While the majority of transport in far-field wakes apparently occurs in the direction propagation of wakes, an interesting feature is characteristic in the near-field in the immediate vicinity of the ship. Namely, sediments resuspended from channel margins and mudflats are transported by strong drawdown currents (up to 2 m s^-1^) associated with the depression wakes. The currents are directed towards the moving vessel and induce a stepwise transport whose integrated effect can eventually be the filling of the navigation channel [27] and increasing the need for dredging to maintain navigation depth.

Our study considered the evolution of the shoreline of the reclaimed area indicated as *Cassa di Colmata B* (CC-B) in Fig 1, located about 200 m from the western margin of the MMC. It has approximately rectangular shape and a surface area of about 3.5 km^2^. The investigated shoreline and beach are 2.3 km long and the approximate foreshore slope is 0.4˗0.6%. The reclaimed area comprises dredged materials, partially mixed with industrial wastes in the northern sector of the study area. The shoreline of the CC-B exhibits an erosion scarp with a variable elevation height above the beach from 0.25 m in the north to 2 m in the south.

## Materials and methods

Analysis of shoreline migration of the west bank of the MMC channel was performed combining long- and short-term data and considering the vegetation line. Seven field surveys were also undertaken over a period of about 10 months to evaluate short-term variations of the erosion scarp. Sediment samples were also collected to characterize the composition and the stratigraphy of the deposits. The long-term investigation was undertaken using a sequence of historical aerial photographs and satellite images, acquired from different sources. All data from the spatial analyses were integrated into a GIS environment to estimate the volume of sediment lost by erosion.

### Field surveys

**Fig 3:**
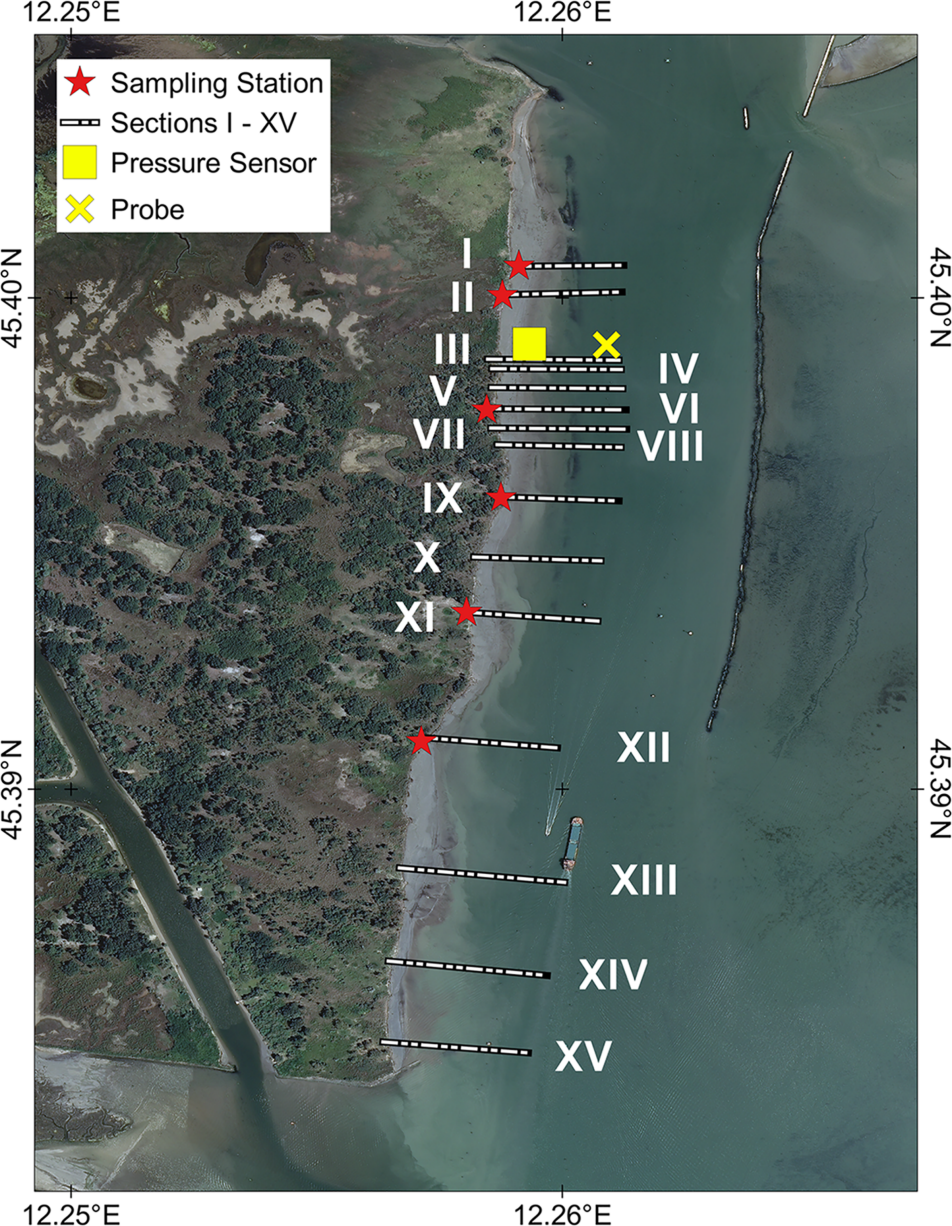
Map of the investigated shoreline. The 15 control sections investigated in the field survey (short term) and in GIS-based analysis (long term) are indicated. Red dots: the location of sampling points for grain-size analysis. Yellow symbols: the location of probe (cross) and pressure sensor (square). Image source: WMS service of The Italian National Geoportal 2012 [48].

The position of the shoreline of the CC-B was measured on seven occasions from April 2014 to January 2015. Fifteen control stations (I˗XV) were established (Fig 3). At each station two poles were positioned along a line perpendicular to the shoreline.

The distance from the landward pole to the edge of the scarp was measured in each survey by tape meter aligning two poles to ensure all measurements were taken along the same line. Due to the fast retreat of the escarpment, a few of the seaward poles were lost after the first survey, resulting in possible minor errors due to having to estimate the line orientation. The technique is affected by some uncertainty (~ 1 cm) but was considered sufficiently reliable given the magnitude of the variations between different measurements. The results of each survey were recorded in a database consisting of tables for each station which integrate numerical and visual information (images taken with a camera Nikon D80 and field sketches) and linked in the GIS system.

An Idronaut Ocean Seven 316 plus probe logger (full-scale 100 dbar, accuracy 0.05% full-scale, resolution 0.002% full scale, sampling depth ~3m, 5 Hz sampling frequency) and a KPSI Model 750 Water Monitor Depth and Temperature Recorder (10 dbar full-scale, precision 0.05% full-scale, ~1m deployment depth, 2 Hz sampling frequency) were used in April 2014 to describe waveforms from vessel wakes. The pressure sensor of the Idronaut probe was zeroed at the sea level before measurements. Absolute pressure measured by the KPSI pressure logger was corrected to water depth using local atmospheric pressure recorded approximately 3 km from the measurement site. Atmospheric pressure compensation was undertaken using KPSI ‘K-ware’ software.

A bathymetric survey of the area between the channel and the shoreline was undertaken in April 2015 using the bottom tracking capability of a ADCP (Teledyne-RDI Workhorse Rio Grande 1200 kHz; depth measurement accuracy 10 mm, resolution 1 mm, working range 0.5˗30 m), mounted on a flat-bottomed boat with transducers at 0.3 m from the surface and used as a single beam echosounder combined with a GPS (Trimble DSM132 DGPS Receiver; update rate 1, 2, 5 and 10 Hz; differential speed 0.1 kn (0.1 MPH, 0.16 km/h, 5.6 cm/sec) accuracy; differential position < 1 m horizontal RMS accuracy with at least 5 satellites, PDOP). The instrument was set to collect georeferenced depth data to a minimum depth of 0.5 m. The acquired raw data were corrected for tide level measured on a tide gauge station located 1 km north of the study area and finally used for the analysis of volume losses. Bathymetric data were processed in the GIS suite QGIS (version 2.16.0).

During field surveys, drone images were occasionally acquired to visualize the processes from an elevation of about 70 m. The drone used was a self-built DJI F450 equipped with a GoPro HERO 4+ stabilized with a dual axes gimbal.

### Grain size analysis

Sediment samples of about 0.5 kg were manually collected at 6 locations along the scarp (Fig 3). Particle-size analysis was conducted in the laboratory in untreated aliquots of about 1 g by laser diffraction (Mastersizer 3000, Malvern) in the range 0.03˗1000 μm. The characteristics of the distribution were obtained using the software GRADISTAT [49].

### GIS analysis

The long-term analysis of shoreline migration for CC-B was performed by analyzing aerial and satellite images for the period 1974˗2015 (S1 Table). The first series of images was acquired in 1974, after the completion of the channel excavation and reclamation of the CC-B. Aerial photographs of the Lagoon of Venice were downloaded from the official geoportal of the Veneto Region [50]. Images were georeferenced using a raster tool included in QGIS (Open Source GIS software. Version 2.10 Pisa). For each aerial image a series of ground control points (GCP) were identified assigning them to a spatial reference system, WGS84/UTM zone 32 N; EPSG:32632 (S1 Table). An orthophoto image taken in 2012 and available in the Web Map Service (WMS) of National Cartographical Portal [48] was used to develop the georeferenced base map by utilizing a 2nd order polynomial as a transformation function and nearest neighbor as the imaging resample method. Root mean square errors (RMS) obtained for GCP transformations during the georeferencing procedure were less than 2 pixels which corresponds to a maximum RMS of 1.6 m when placed in map units. Images used for the investigation of the shoreline evolution have a maximum RMS of 1.1 m. Recent imagery from the period 2009˗2015, available as georeferenced images, were loaded in GIS environment with the plugin tool *Openlayers* from web services of Google (Digital Globe) and Microsoft ® Bing ™ Maps and only used for elaboration.

The shoreline position was identified for each available image and used in the GIS environment to trace its migration. The vegetation line, a common indicator of shoreline position employed in the literature [51], was used for the analysis of erosion trends. The marked difference between the vegetation cover of the CC-B and the fine sand deposit of the beach makes shoreline identification with good precision possible due to the abrupt change at the erosion scarp. In the aerial images acquired in 1974, the shoreline is assumed to be the bulkheaded edge of CC-B.

In order to measure differences in the shoreline position in subsequent images, calculate the rates of retreat, and analyze spatial and temporal variability of the erosion process in the period covered by the available imagery, the same 15 control sections discussed above were used in the GIS environment (Fig 3). Distances were calculated from the intersections of the shoreline on each image with the selected control sections.

The volume of sediment eroded from the CC-B was estimated from the differences between two digital terrain models (DTM) from 1968 to 2015. The reference base for the DTM is the official 2007 DTM available on the Geoportal of the Veneto Region [50]. It is reasonable to assume that this base is representative of the elevation of the sediment surface as it was after the reclamation of the area.

The 1968 DTM data were obtained by integrating in the 2007 DTM the elevation of the original bulkheaded shoreline. This dataset is the only information reported on the published topographic maps of 1968 [50]. This elevation was attributed to the whole of the missing area. The assumption is justified by the relatively flat morphology visible in 1974 aerial images of CC-B. Clouds of points obtained by merging the two datasets were interpolated using the Thin Plate Spline function in SAGA 2.1.2 GIS software. The new raster elevation base map obtained for the 1968 DTM has an accuracy of 10 meters which can be considered of minor importance given the magnitude of the observed changes.

The 2015 DTM was instead obtained by cutting the 2007 DTM (spatial accuracy 2 m) along the 2015 shoreline, obtained from the latest satellite image, and merging the elevation of the remaining area, with bathymetries of the eroded area acquired during the survey of April 2015.

## Results

**Fig 4:**
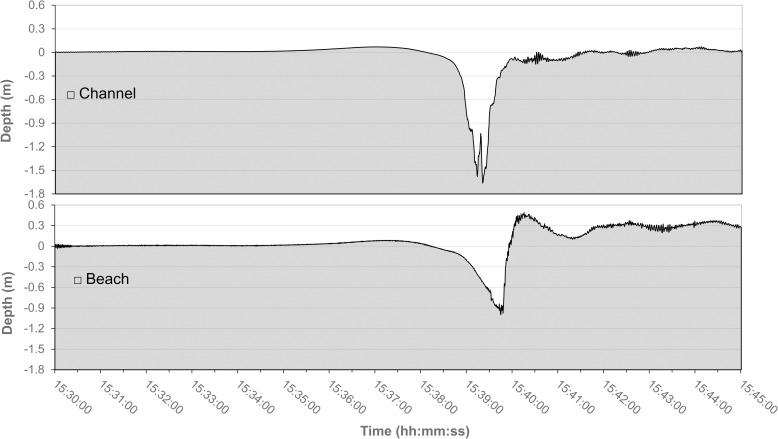
Ship induced depression wake. A depression wake originated from the cargo ship Abu Dhabi Star on 1^St^ April 2014 at 03.30 PM in transit along the MMC as measured on the channel side at 70 m (top panel), and over the CC-B shore, at about 190 m (bottom panel) from the channel center.

A typical ship-induced depression wake has a characteristic symmetric V-shape in the navigation channel and becomes highly asymmetric over the shoals. Fig 4 shows the water level measured by pressure sensors located on the west side of the MMC and on the beach shore at a distance of 70 and 190 m from the channel center during the passage of a vessel. The asymmetry of the wake over the shallow water area implies high near-bottom velocities on the steep trailing slope (up to 2.0 m/s) and the resulting high shear stress can induce sediment resuspension [25].

**Fig 5:**
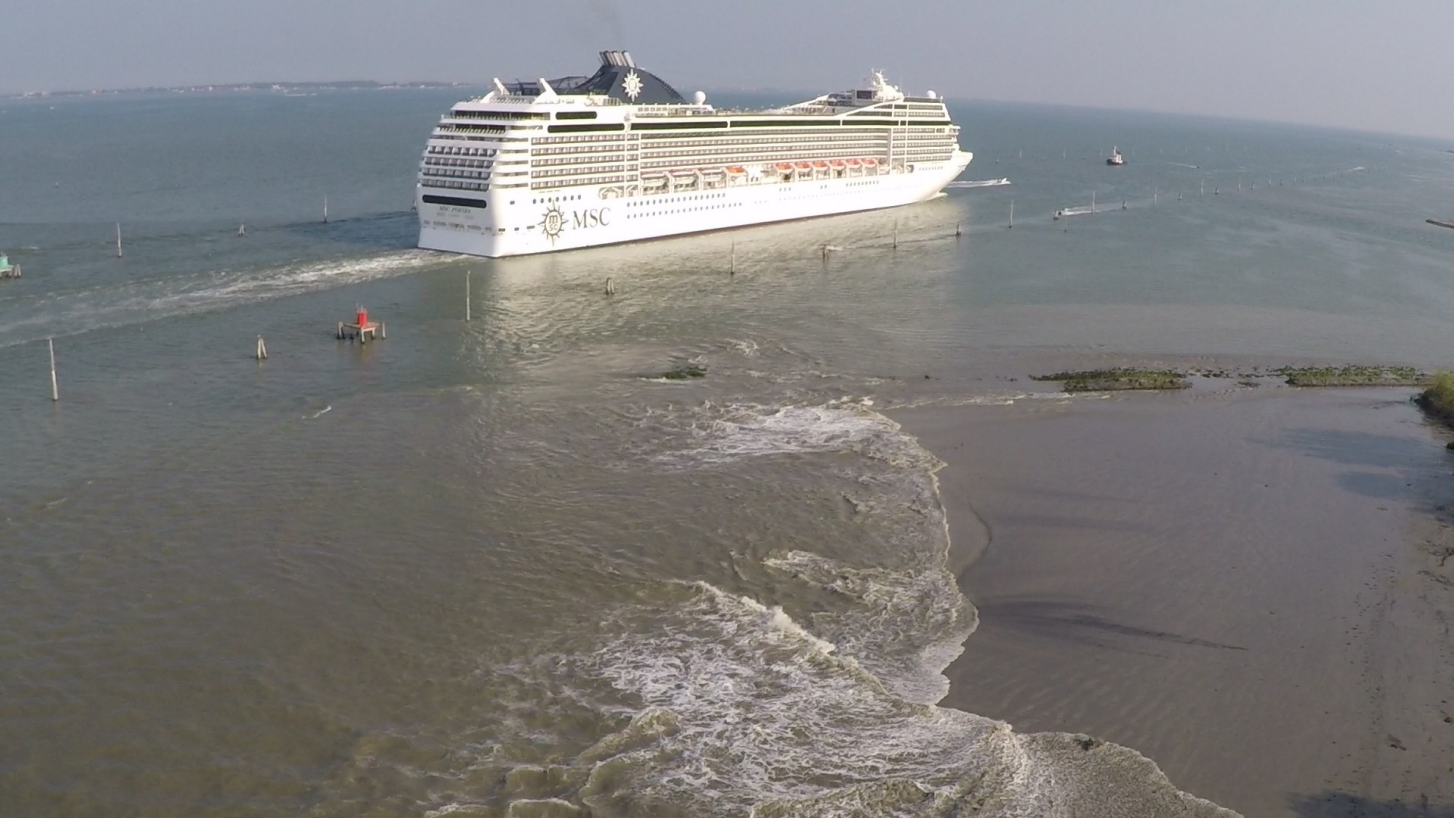
Aerial view of a cruise ship transit in the MMC. The depression wake exposes the collapsed materials of the original rip-rap revetment of the embankment at the southern end of the *Cassa di Colmata B* (CC-B). Image source: author’s picture.

On the shore, a typical wake starts with a retreat of the water which progressively accelerates towards the channel. This rapid flow with a speed up to 2 m/s, estimated from movies of the wakes taken by the drone, is able to move large amounts of sediment. Immediately after reaching the minimum level, following the passage of the ship, the water returns with a steep breaking wave which moves parallel to the coastline at the highest part of the beach. This entity transports large amounts of sediment in the alongshore direction (Fig 5). At this time the behavior of the fluid is closer to a mass transport phenomena than to typical transport processes in water, and the high density of the fluid (sediment concentration can easily exceed 100–150 g/l [25, 27]) also implies a much larger erosive potential of the moving mass. The evolution of a typical event is clearly visible on the drone image in Fig 5, taken after the passage of a cruise ship in the MMC.

**Fig 6:**
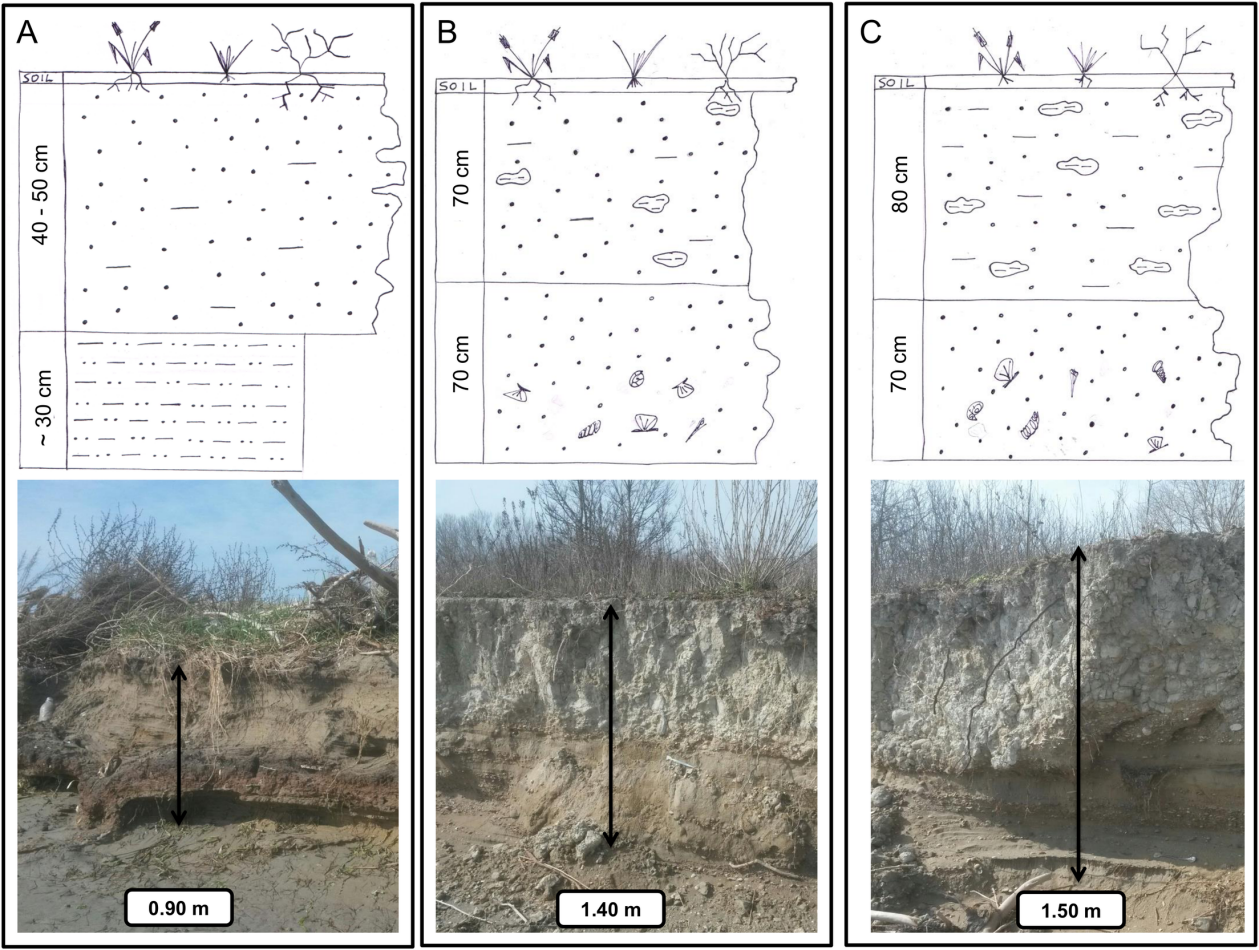
Typologies of stratigraphic sequences in the CC-B. A) northern section, bauxite deposits at the bottom, overlaid by sandy sediments from dredging; B) central section, the bottom is a composite layer, mostly sandy sediments, with lenses of shells and shell fragments overlaid by a silty-sand layer hosting clods of mud deriving from dredging; C) southern section, the bottom layer is a natural sandy sediments from fluvial deposition (former Brenta River) overlaid by dredged mud with a large amount of mud clods.

**Fig 7:**
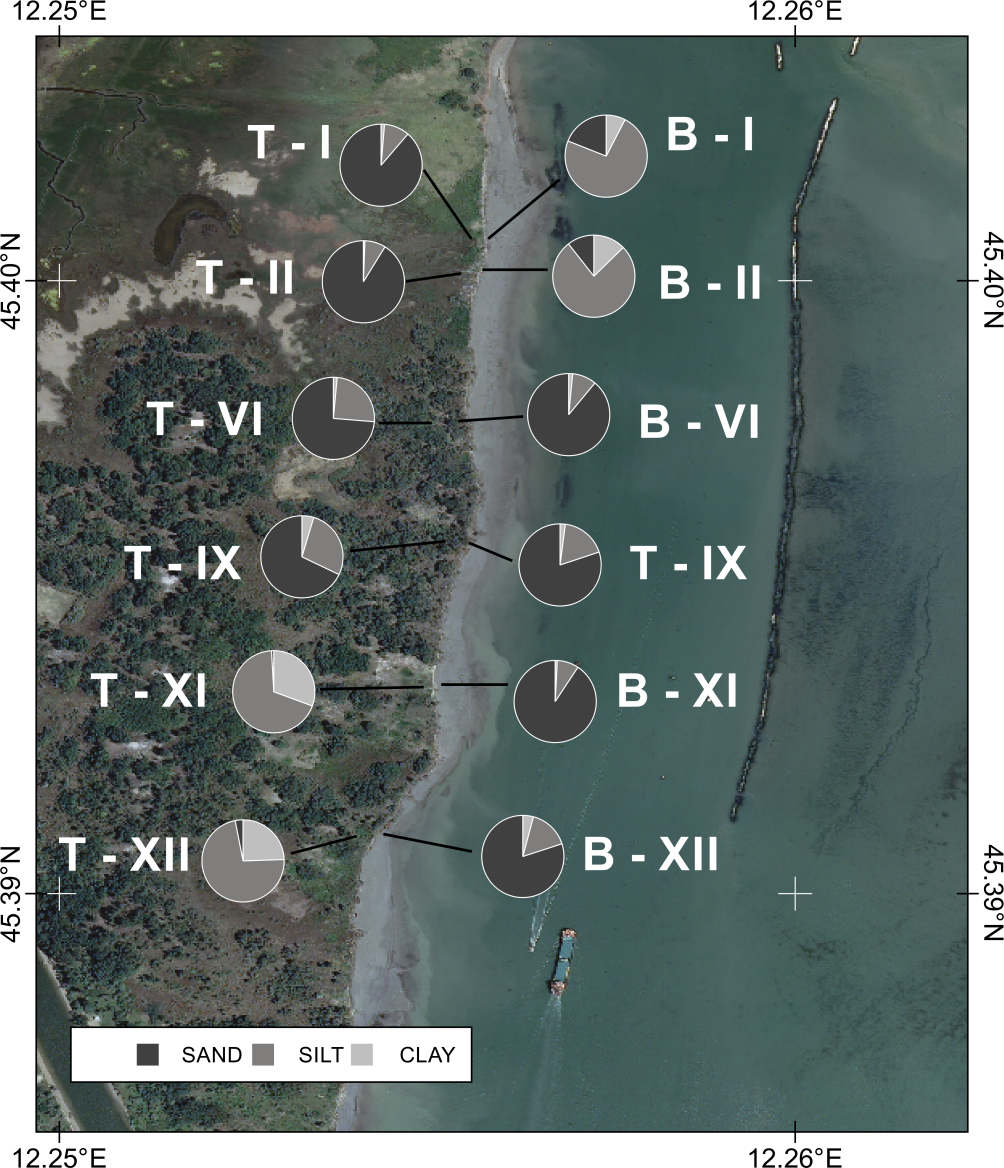
Sediment grain size distribution. Samples collected on the main erosional scarp of the CC-B. T = top of the scarp; B = bottom of the scarp. The numbers correspond to the fifteen control sections investigated in the field survey. Image source: WMS service of the Italian National Geoportal 2012 [39].

These high-energy processes acting on the shore affect the morphology of the beach and the margins of CC-B, which is a typical erosion scarp. As a result of the overlapping of natural lagoon sediment with dredged materials, the sedimentological characteristics of the CC-B are quite complex and variable, as shown in Fig 6 and Fig 7 where the results of grain-size distribution analyses are respectively shown (S2 Table).

To the north (Fig 6A) bauxitic deposits from industrial aluminum processing of clayey-silt composition are present at the bottom of the sequence and are overlaid with predominantly sandy sediments derived from dredging. In the central sector (Fig 6B) the bottom is a composite of mostly sandy sediments with layers and lenses of shells and shell fragments and is overlaid with a silty-sand layer locally hosting clods of compacted mud deriving from dredging. In the southern part of CC-B deposit the bottom (Fig 6C) is a natural sandy sediment layer, with local shell deposits. This material is a product of the fluvial deposition by the former Brenta River flowing in this area [52] until the diversion operated in historical time (mid-1500s) by the Venetian Republic. Similarly to the central area, this layer is overlaid by the dredged deposits, mostly mud, with a larger number of compact mud clods. The materials of the reclaimed layer (top layer in Fig 6) in the central and southern sectors originated from the deep dredging of the channel. This waterway was cut through layers ranging from land derived deposits of the late Pleistocene to more recent Holocene lagoonal deposits [53]. The variable texture of the original bedrock and the dredging process determined the final structure of the reclaimed materials as observed in the field. The clods are originally fragments of deep layers cut by dredger tools while the matrix is comprised of the same materials, transported as dredging slurry during the reclamation works. Over all of the CC-B, the stratigraphic sequence is covered by a vegetated soil varying in thickness from 50 to 100 mm.

The sediments found on the shore at the base of the erosion escarpment are mostly comprised of fine and very fine sand with a minor percentage of mud (less than 6%) and are characterized by ripples perpendicular to the shoreline. Furthermore, ellipsoidal mud boulders of varying sizes (from 20 to 200 mm) can be found on the shore surface in the central and southern sections. These boulders are the product of the reshaping of mud clods, collapsed from the CC-B escarpment, by the rolling action of the strong heavy-density currents associated with ship wakes.

The collapse of the scarp is caused by undercutting and erosion of the silt-sandy layer at base of the deposits, as shown in Fig 6B. This layer is more susceptible to erosion than the overlying silt-clay deposits. Collapsed materials accumulate over the beach surface at the foot of the scarp causing a temporary reduction in the rate of retreat until complete removal by the repeated action of ship wakes.

### Short term analysis: Field surveys

**Fig 8:**
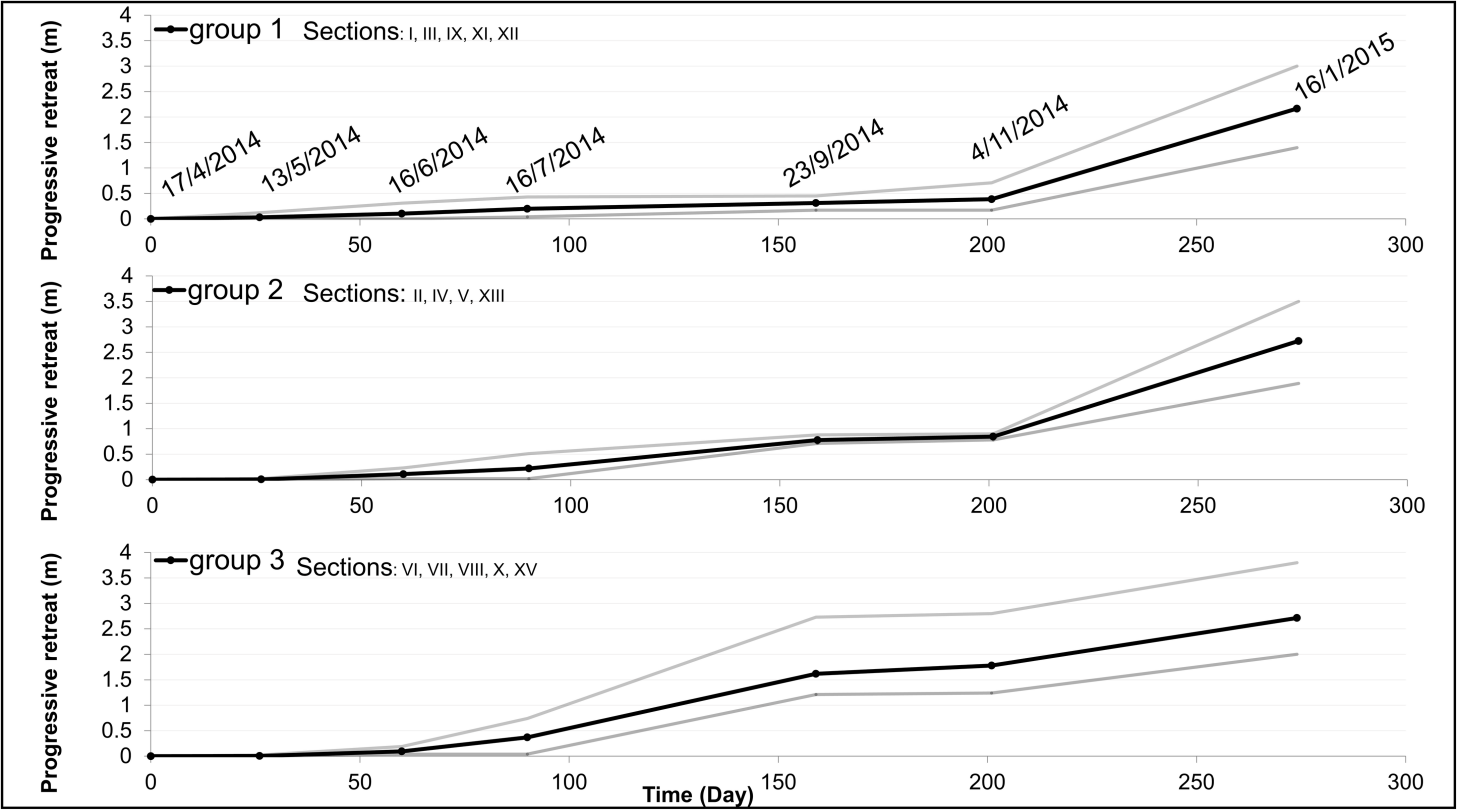
Cumulative retreat of the CC-B shoreline from April 2014 and January 2015. The fourteen sections have been clustered in the three main groups. Average trend for each group is shown together with the range of variation (grey lines).

The analysis of the results of field investigations conducted from April 2014 to January 2015 showed the rate of shoreline retreat over a period of approximately 9 months (S3 Table). We observed a generally large variability in retreat with no apparent spatial differentiation. The measured erosion ranged from 1.4 m, in section III, located in the northern sector, to 3.8 m in section VII, located in the central-northern sector. We can cluster the behavior of the different sections in three main groups (1~3), as shown in (Fig 8) where the average trend for each group is shown together with the range of variation. In sections of group 1 erosion occurs slowly, less than 0.5 m, in the first 200 days and increases rapidly in the following period, until the last survey, reaching a final value of 2.2 m. Sections of group 2 undergo a retreat up to ~ 0.8 m in the first 159 days, followed by an almost unchanged situation lasting till day 200 and a final marked increase in erosion rate in the last period, leading to a total retreat of ~2.7 m. Group 3 is finally characterized by a more regular recession of the escarpment which starts from day 60 (~ 0.1 m) and ends at ~ 2.7 m.

In most sections the recession rates are quite variable: pulses of retreat are followed by periods of relative stability, which are not synchronous. Other stations (II, VI-VIII) are characterized by a more regular rate of erosion.

Data from section XIV were excluded as both reference poles were lost during an extensive burst of erosion occurred after the first survey of May 2014.

### Long term analysis: Remote sensing and GIS

**Fig 9:**
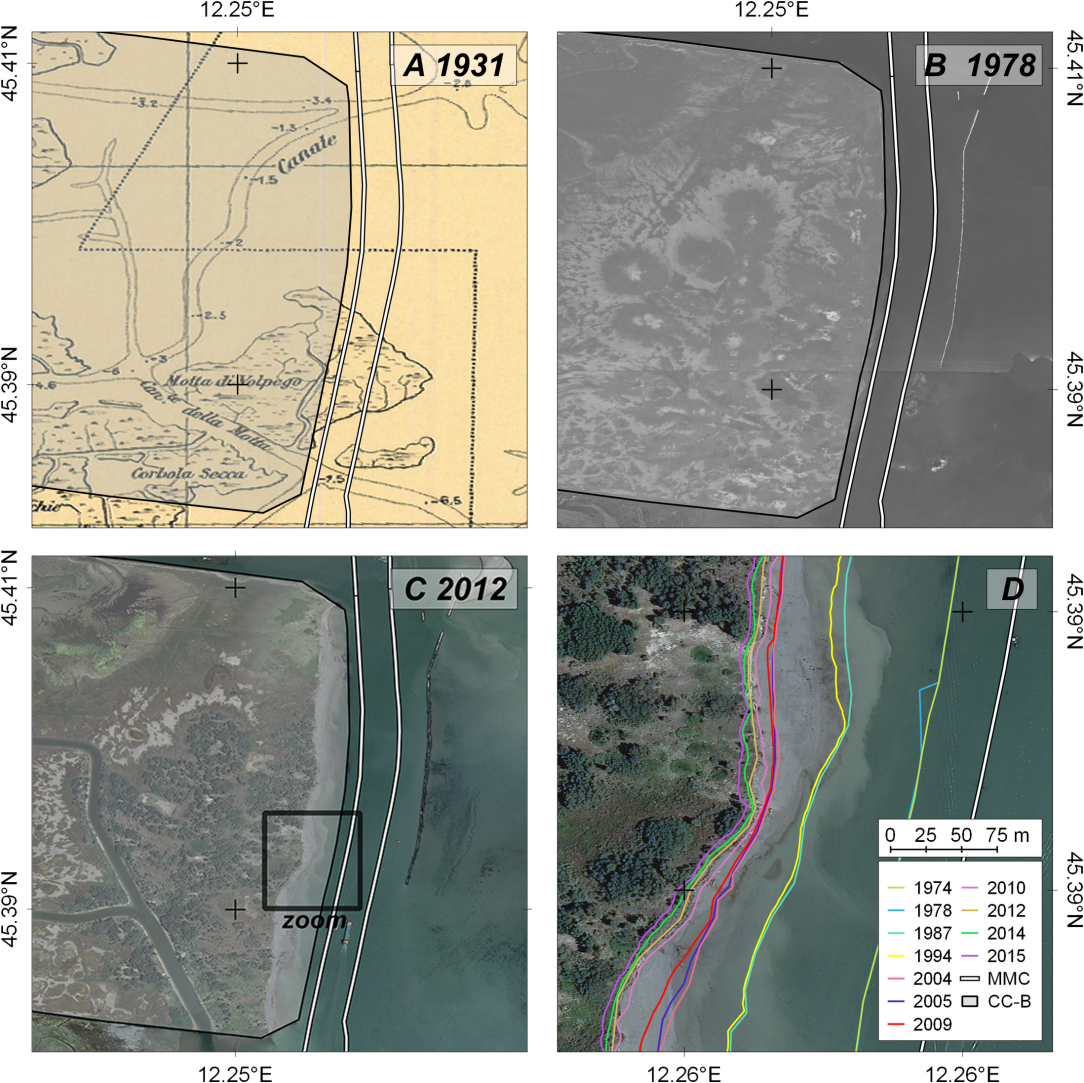
Long-term evolution of the study area. The outline of the original extension of CC-B is shaded in the three images (A, B, and C); the lower right enlargement (D) shows the system as it appeared in 2012 and the evolution of the shoreline from 1974 to 2015. The MMC margins are marked with white lines. Image source: (A) open database CIGNO [39]; (B) Veneto Region geoportal [50]; (C and enlargement D) WMS service of the Italian National geoportal [48].

The first images available for the analysis of the long term modifications of the CC-B margins date back to 1974. Previous aerial views show the area as it was in 1954, before the construction of the navigation channel and the reclaimed areas. Other information on the pre-existing morphology can be found in the old topographic maps. Three examples of the georeferenced data used for the long term investigation are shown in Fig 9.

The outline of the reclaimed area, as it was after its construction is shaded in all images. The historic map of 1931 [39] shows the pristine morphology of the area (Fig 9A), as it also appears on the first available aerial images of 1954. In the map, large extents of salt marshes and mudflats are visible. The sequence of images shows the loss of the original morphology and the consequent loss of habitats which was suggested by the study of the bathymetric evolution of the central lagoon [47]. The enlargement of the study area (Fig 9D) shows the progressive evolution of the shoreline, obtained from all the available imagery, traced by color-coded lines. The maximum total shoreline retreat is 163 m in section XII and the average erosion rate is therefore in the order of 3˗4 m/yr.

**Fig 10:**
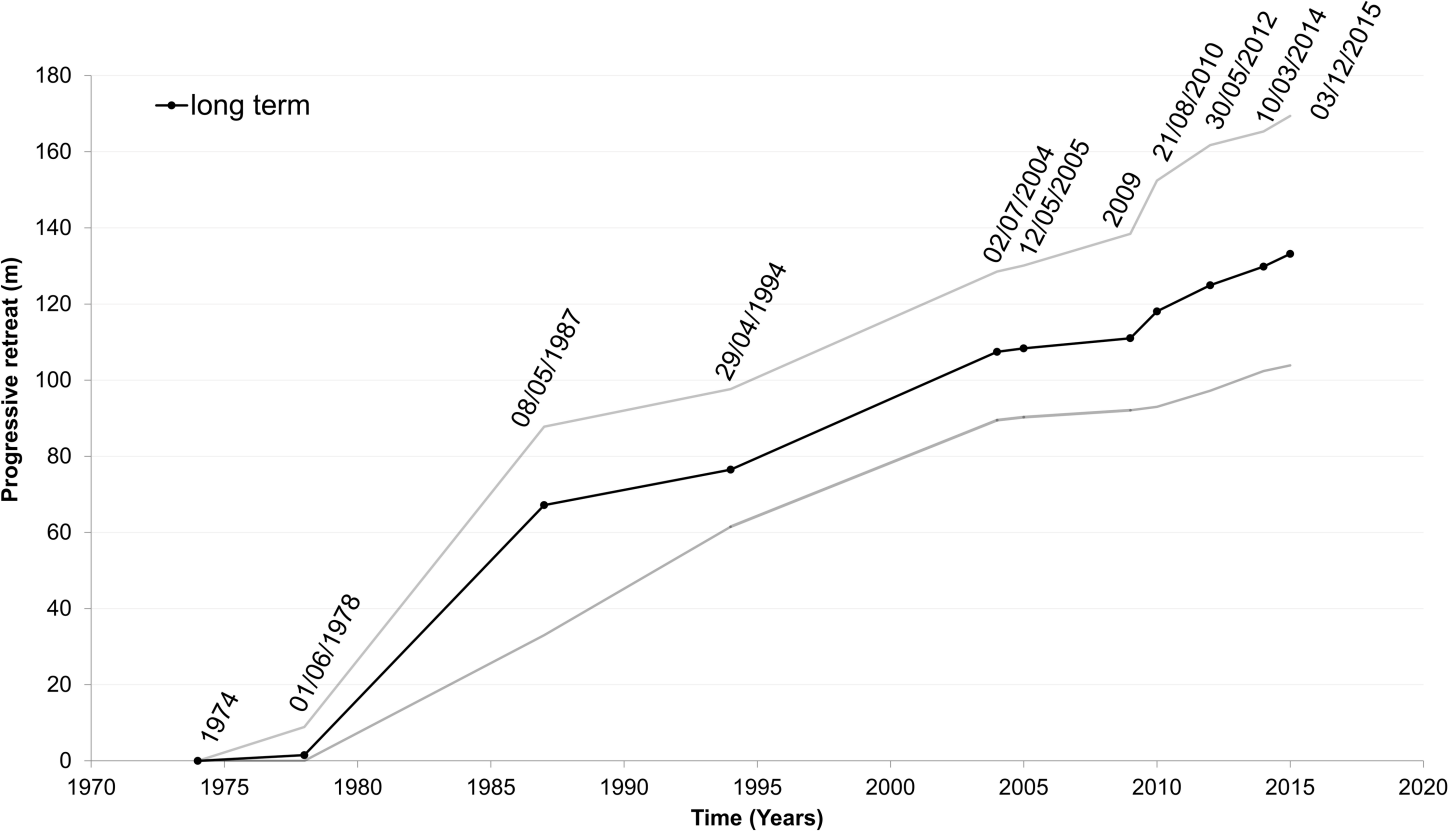
Cumulative change in CC-B shoreline position in the period 1974–2015. Data are calculated from the shoreline of 1974. The variations of the shoreline were obtained by detailed GIS-based analysis of the fifteen control sections. Grey lines indicate the range of variation.

For each of the control sections we analyzed the progressive changes of the shoreline within the GIS environment (S4 Table). The results are summarized in Fig 10 where the progressive change in the shoreline position is plotted versus time taking as reference the shoreline of 1974.

There is no significant change between 1974 and 1978, and in this interval the erosion process only acted on the shoreline protection causing its failure. After 1978, presumably once the bulkhead protection was dismantled, the shoreline retreat became faster and proceeded with a comparable rate in all sections. The largest changes occurred during the period from 1978˗1987. During this period the change ranges from about 19 to more than 80 m, which corresponds to regression rates from about 2 to about 9 m/yr.

**Fig 11:**
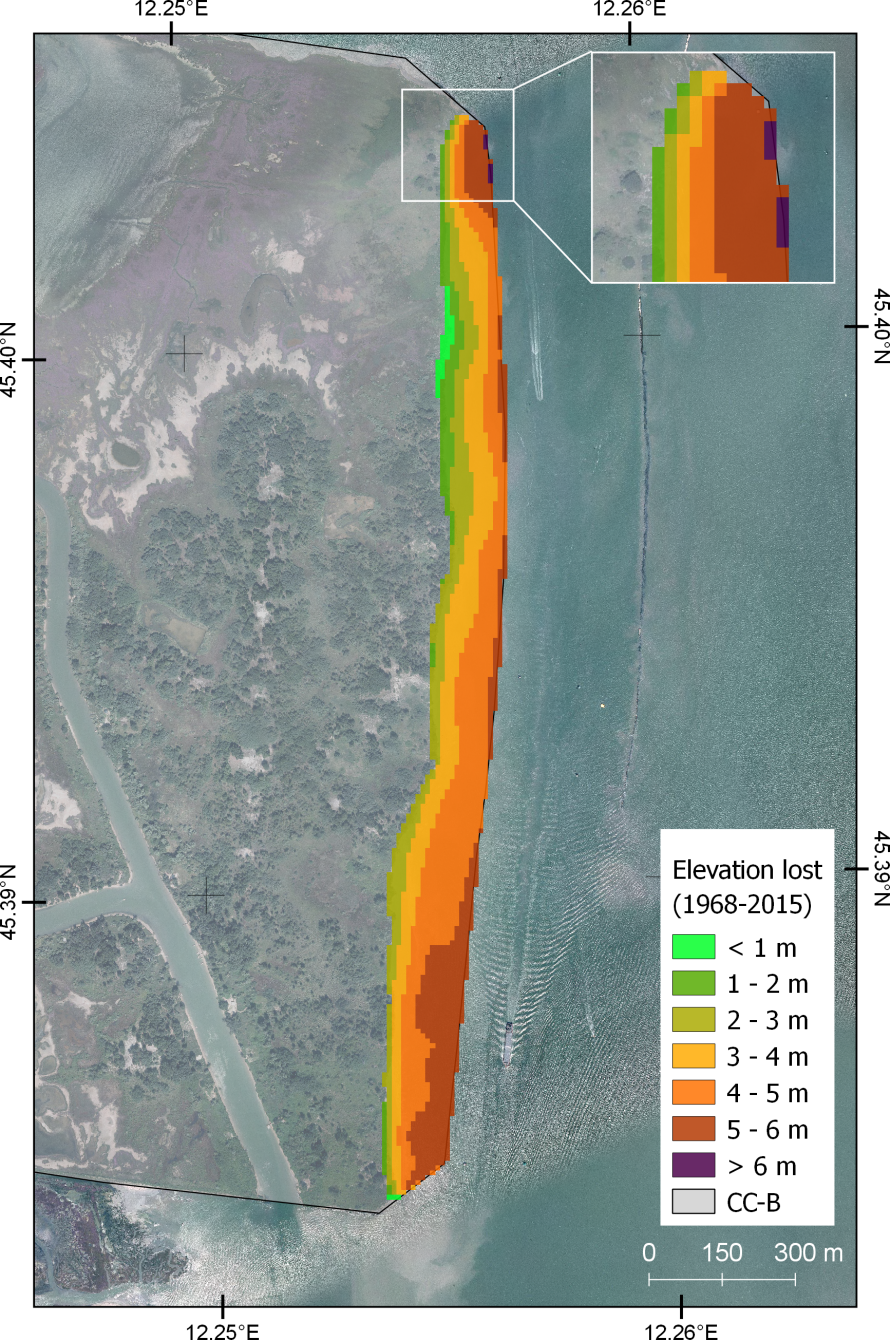
Changes in the elevation of CC-B reclaimed area. The differences in elevation were obtained from 1968 and 2015 DTM. Inset: enlargement of the northern part of the studied area, where the maximum erosion was estimated. Background image source: WMS service of the Italian National Geoportal 2012 [48].

The analysis performed within the GIS environment enabled the calculation of the amount of sediment eroded from CC-B over the last four decades. The loss of material calculated from the difference between the 1968 DTM and 2015 DTM is represented in Fig 11. A considerable loss affected the CC-B shoreline and the total surface eroded in the investigated period amounts to 31.4 ha. The maximum vertical change caused by erosion of the channel side (~ 6 m) is in the northern sector of the CC-B. However, the largest extension of the erosion area is in the central-southern sector which underwent a significant loss in the period 2009˗2010. The total volume lost is about 1.19×10^6^ m^3^, corresponding to a loss of 3.0×10^4^ m^3^/yr.

## Discussion

A significant proportion of the vessels in transit along the MMC generate large depression waves which can potentially cause significant sediment resuspension and erosion of the channel sides and mudflats [23, 27].

The contribution to the total erosion measured in the study site by natural forcings in the lagoon can be considered of minor importance compared to the effects of ship traffic. The frequency and fetch of wind with high intensity is relatively low: less than 10 events per year are strong enough to erode the shoreline of this area. The predominant wind speed and direction does not support erosion events. Tidal currents in this part of the lagoon are typically slow, around 0.1˗0.2 m/s. On the other hand, recent research work [54] reported a total of 380 ship wakes higher than 0.4 m in a series of 589 wake systems recorded over a time interval of 45 days in April-May 2016. As commercial traffic is consistent year-round, this accounts for approximately 3000 events with significant energy on a yearly basis. In terms of frequency and magnitude of its effects, vessel traffic in the MMC can then significantly affect the morphology of the areas surrounding the navigation channel.

**Fig 12:**
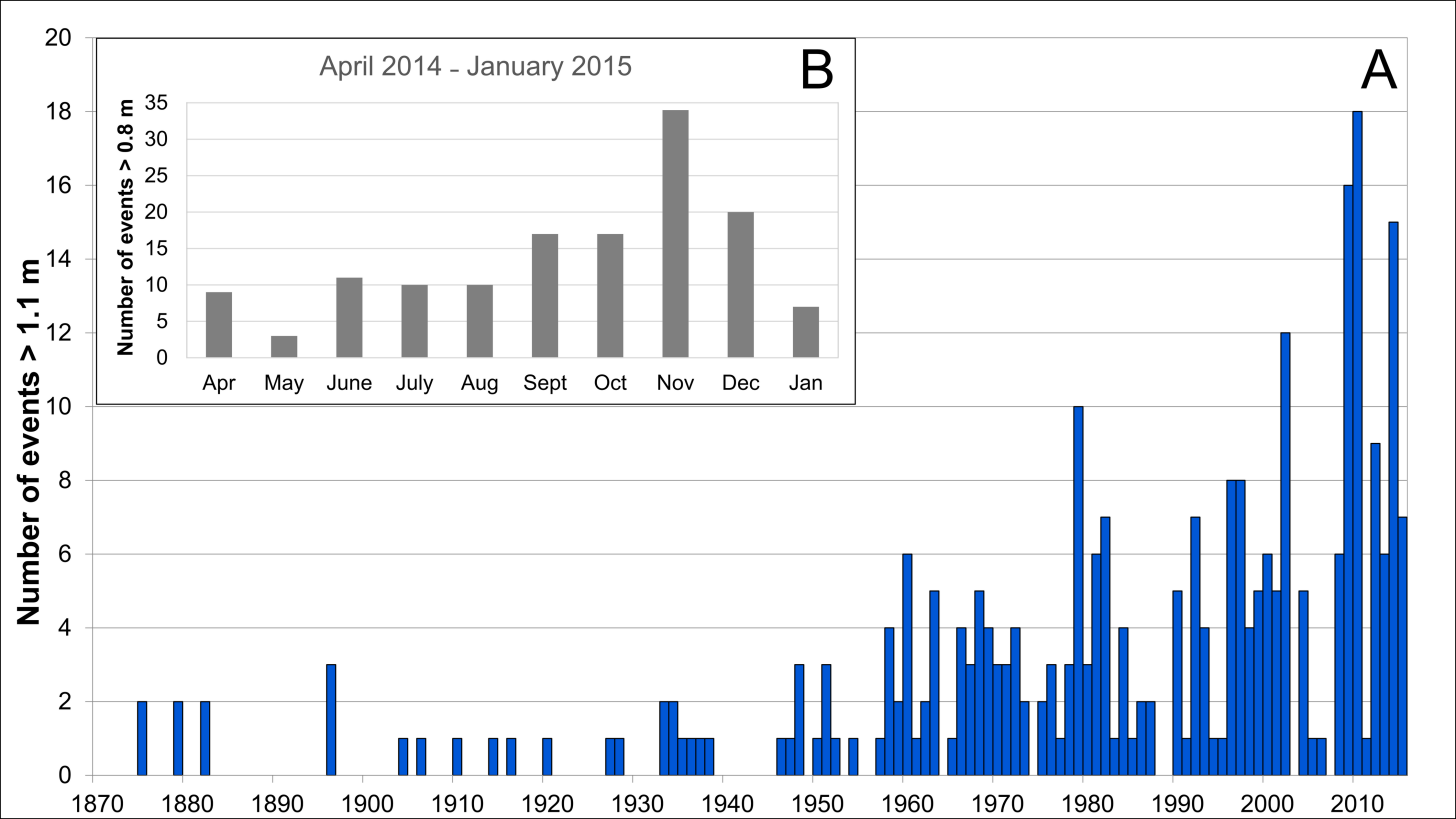
High tides in Venice. Annual distribution of high tides ≥1.1 m recorded in Venice from 1872 to 2015 (A). The inset shows the distribution of tides > 0.8 m in the period April 2014-January 2015. (B). Data courtesy Istituzione Centro Previsione e Segnalazione Maree of the Municipality of Venice [55].

The process of shoreline erosion induced by ship wakes on the CC-B is variable in space and time, as shown by the results of short- and long-term investigations. The retreat depends on the characteristics of sediments, stratigraphy, morphology, vegetation cover of the existing landforms and on the tidal levels. Our results combined with official tide data show that the maximum erosion rate occurred in conjunction with higher frequency of high tides or storm surges (*Acqua Alta*). In Fig 12A the annual distribution of high tides (>1.1 m) between 1872 and 2015 is shown [55]. From an initial frequency of one exceptional high tide per year, at the beginning of the last century, the average frequency has risen to 9˗10 high tides per year in the more recent period. The annual frequency of exceptional tidal levels underwent a large increase from 1960. The highest erosion rate in 2009˗2010 is linked to the increased frequency of high tides that occurred in that period, reaching 16 and 18 events respectively (Fig 12A). Moreover, from the results of the short term analysis we observed the maximum erosion during the months with larger frequency of high tides (> 0.8 m), from November 2014 through January 2015 (Fig 12B).

The observed evolution shows that from the time the CC-B reclamation was completed the area was subjected to increasing stress from the combination of ship wakes and higher tidal levels which determined an immediate start of the erosion processes, though slowed in its initial phases by the presence of the rip-rap protection.

The collapse of the scarp, combined with the higher water levels, explains much of the spatial-temporal variability observed in the short-term. After the failure of slabs of the CC-B edge, the debris accumulates over the beach at escarpment base and acts as a temporary shoreline protection, until complete removal by ship-wake associated currents. In the short-term, the erosion process is an intermittent movement which proceeds irregularly as a result of the spatial changes in the textural and rheological properties of the reclaimed materials.

The correspondence between short- and long-term shoreline retreat also suggests that the cross-section of the navigation channel and its margins have not yet reached a stable profile and are still adapting to the impacts of traffic. The port traffic has increased over time both in size and number of vessels, likely preventing an equilibrium being reached, and the channel section is still adapting. Rapaglia et al. [25] studied the effects of the depression waves on the mudflat at east of the channel. They found that wakes of this type can cause significant sediment resuspension at a distance of >300 m from the channel. The results of theoretical models by Rodin et al. [29] showed that the wakes maintain significant heights for even larger distances from channel margins.

Tate et al. [56] modeled vessel traffic in a channel surrounded by shoals and found that ship induced circulation can suspend sediment in the channel and adjacent shallow-water areas eventually causing channel shoaling. Similar effects of ship wakes were observed in the MMC by Gelinas et al. [28] and Rapaglia et al. [27]. These authors showed that the passage of vessels in the channel and the dynamics of the associated depression waves can cause the resuspension of large amount of sediments from the channel sides and adjacent mudflats. According to their results, these materials are transported towards the channel during every ship passage. The resulting effect of frequent transits is a stepwise movement of sediments from the mudflats to the channel.

The progressive erosion of the channel margins and, more generally, of the mudflats can therefore cause the accumulation of the eroded sediments on the channel bottom. In the long term, the described process can be responsible for a significant proportion of the total volume lost by the central lagoon basin after the construction of the MMC waterway [47]. The net sediment flux calculated for the recent period through the Malamocco inlet is in fact considerably lower than the flux through the other two inlets [57], so that we can reasonably assume that most of the sediments currently mobilized from mudflats eventually redeposit into the MMC and other natural tidal channels of the central lagoon basin leading to aggradation. Erosion and channel aggradation is also documented in similar contexts worldwide. According to Rosati et al. [58], dredging interventions historically performed in the Galveston-Houston channel, U.S.A., to increase the section width and depth, always led to further aggradation of the channel bed, presumably at the expense of the shallows. Although the ship wakes and their effects were not investigated in detail by these authors, there are recent visual evidences of high ship wakes in the area, similar to those studied in the Lagoon of Venice. Townsend et al. [16] described shoals developing in the Gulf Intracoastal Waterway, U.S.A., as a result of the erosion of channel margins. Their estimates of erosion rates of the channel margins are comparable to those determined in this study. In the Elbe River, near the port of Hamburg, where high depression waves are frequently observed during the passage of large vessels, there is visual evidence of erosion, and groins have been built in some locations to protect the shoreline from sediment removal.

In the Lagoon of Venice, the progressive depth reduction of the channel results in an increased cost of maintenance dredging. According to data published by the local Port Authority [59], the costs of dredging and disposal of about 800.000 m^3^ of sediment from shipping channels is 40 million Euro. Assuming that the volume lost by CC-B is all deposited in the navigation channel we can calculate a dredging cost of 1.5 million Euro per year or 60 million Euro for the 40 years period considered in this study (at today’s values). However, dredging is not the only cost associated to the morphological changes. The consistent loss of habitat associated to the erosion of channel margins and mudflats has an impact on the ecosystems services, which needs to be evaluated.Moreover, the extensive deepening of the central lagoon basin also had consequences on its general hydrodynamics, increasing its vulnerability to the effects of storm waves and exceptional high tides with possible feedbacks to the costs of storm-surge protection measures.

Contamination is another important consideration related to the erosion of the CC-B and the other reclaimed channel margins. These artificial landforms were created with a mixture of dredging materials and industrial wastes and their erosion has caused the release of contaminants into the environment. In the northern part of the CC-B the red muds consisting of bauxite residues from aluminum processing are frequently resuspended and mobilized by ship-wake associated currents. These and other by-products of industrial processes have long contaminated the sediments of the area [60]. For this reason the Porto Marghera Industrial Zone was designated as a contaminated site of national interest (SIN, Italian law 426/1998). Large areas of the lagoon near the PMIZ have high concentration of organic and inorganic contaminants in the sediments [60–65]. As suggested by Rapaglia et al. [27], these contaminants can be remobilized by the frequent resuspension and the consequent reaeration of bottom sediments with potential harmful effects on the whole ecosystem of the central Lagoon of Venice.

## Conclusions

The design and management of waterways in shallow water coastal systems is undoubtedly a challenge. Besides other environmental impacts, the effects of ship traffic can significantly affect the morphology of the channel margins and the surrounding shallow water areas, as shown by this case study.

From the results of our investigation it follows that a few decades of traffic in the Venice Lagoon caused extensive shoreline retreat along the waterway. The maximum recession over four decades is ~160 m and regression rates up to 4 m/y. The total volume lost from the studied system is 1.19×10^6^ m^3^ corresponding to 3.0×10^4^ m^3^/y most of which consists of protected areas for the conservation of fauna.

The progressive erosion of the channel margins and of the adjacent mudflats determines the accumulation of the mobilized sediments on the channel bottom, increasing the frequency and cost of maintenance dredging. However, the economic implications of the erosion are not the only undesirable effect. Firstly, in the long term, the process can be responsible for a significant proportion of the total loss of habitat observed in the central lagoon basin with consequences on ecosystem services. Secondly the progressive deepening of the central lagoon basin also has consequences on the general hydrodynamics of the lagoon, increasing its vulnerability to the effects of storm waves and exceptional high tides. Finally, the continuous sediment mobilization and re-aeration can mobilize contaminants into the water column, with potentially harmful effects on the whole lagoon ecosystem.

Although it is hard to imagine a strong pressure, such as that of modern international port facility, without significant impacts on the environment, a conceptual model which considers the effects of the described processes, would surely permit the management of vessel traffic in a sustainable way minimizing the impacts on the morphology and on the ecosystem.

## Supporting information

S1 Table Characteristics of the georeferenced images used for the GIS analysis of the shoreline evolution. Columns n.GCP and RMS report the number of ground control data points and root mean square error in pixels.
(XLSX)

S2 Table Results of the grain-size analysis. Grain size distribution (in volumetric percentage) of sediment samples.
(XLSX)

S3 Table Cumulative retreat in the short-term. Values of cumulative retreat of the CC-B shoreline from April 2014 to January 2015. Data from section XIV were excluded as both reference poles were lost during an extensive burst of erosion occurred after the first survey of May 2014.
(XLSX)

S4 Table Cumulative retreat in the long-term. Values of cumulative change in the CC-B shoreline position in the period 1974 to 2015.
(XLSX)

S1 Appendix License permission for aerial and satellite imagery. Request forms and granted license permissions for aerial and satellite images displayed in the text.
(PDF)
