# Supplementary material for: Fast shoreline erosion induced by ship wakes in a coastal lagoon: Field evidence and remote sensing analysis
Source: PLoS One. 2017 Oct 31;12(10):e0187210. doi: 10.1371/journal.pone.0187210 (PMC5663627; doi:10.1371/journal.pone.0187210)
Supplement: S1 Appendix — Request forms and granted license permissions for aerial and satellite images displayed in the text. (PDF) [file pone.0187210.s005.pdf]

REGIONE DEL VENETO

giunta regionale

Data 22/09/2017

Oggetto: Autorizzazione alla pubblicazione. Authorization to publish.

Spett.le/To:
Dr. Luca Zaggia
Consiglio Nazionale delle Ricerche
ISMAR - Istituto di Scienze Marine
Arsenale-Tesa 104, Castello 2737/F – 30122
Venezia, Italy

Egr. Dr. Luca Zaggia,

In risposta alla vostra richiesta del 21 settembre 2017, vi autorizziamo alla pubblicazione nella rivista scientifica PLOSONE dell'immagine tratta volo REVEN 1978 del WMS Service del Geoportale Regionale Veneto, in base alla "Creative Commons Attribution License" (CCAL) CC-BY4.0. (http://creativecommons.org/licenses/by/4.0/).
L'autorizzazione è concessa per i fotogrammi specificati nella vostra richiesta: strisciata 10, fotogramma 314; strisciata 11, fotogramma 243.

Dear Dr. Luca Zaggia,
Following your inquiry of September 12th 2017, we grant you the permission to publish on the scientific journal PLOSONE the image from the flight REVEN 1978 of the WMS service of the Geoportal of Veneto Region, under the Creative Commons Attribution License (CCAL) CC BY 4.0 (http://creativecommons.org/licenses/by/4.0/).
The permission is granted for the aerial photos specified in your request letter (strip 10, photo 314; strip 11 photo 243).
Best Regards,

Cordiali saluti.

P.O. Database geotopogrfico, reti geodetiche e centro documentazione per la cartografia
Il Titolare
Dott. Umberto Trivelloni

Area Tutela e Sviluppo del Territorio
Direzione Pianificazione Territoriale
Calle Priuli, Cannaregio 99 – 30121 Venezia - tel. 0412792139 – fax 0412792383
pianificazioneterritoriale@regione.veneto.it
pianificazioneterritoriale@pec.regione.veneto.it

Cod. Fisc. 80007580279 *Codice Univoco Ufficio* **C8UYLY** *P.IVA 02392630279*

REFERENDUM SULL' AUTONOMIA DEL VENETO 22 ottobre 2017
I seggi saranno aperti dalle ore 7.00 alle ore 23.00

Ministero dell'Ambiente
e della Tutela del Territorio e del Mare

DIREZIONE GENERALE
PER LA SALVAGUARDIA DEL TERRITORIO E DELLE ACQUE

IL DIRETTORE GENERALE

MINISTERO DELL'AMBIENTE E DELLA TUTELA
DEL TERRITORIO E DEL MARE
Direzione Generale per la Salvaguardia del Territorio e delle Acque

REGISTRO UFFICIALE - USCITA
Prot. **0020025**/STA del **22/09/2017**

Al Dott. Luca Zaggia
Istituto di Scienze Marine - Consiglio Nazionale delle ricerche
luca.zaggia@ve.ismar.cnr.it
segreteria@ve.ismar.cnr.it

Oggetto: Autorizzazione alla pubblicazione figure da dati GN nella rivista scientifica PLOSONE .

Facendo seguito alla richiesta allegata, acquisita al protocollo della scrivente Direzione Generale con protocollo n. 19970/STA del 22 settembre 2017, si autorizza, ai sensi della licenza "Creative Commons Attribution License" (CCAL) CC-BY4.0, la pubblicazione delle immagini utilizzate nell'articolo "Fast shoreline erosion induced by ship wakes in a coastal lagoon: field evidence and remote sensing analysis" derivate dal Servizio WMS denominato "Ortofoto a colori anno 2012 con relative date del volo", pubblicato dal Geoportale Nazionale (http://www.pcn.minambiente.it/mattm/).

L'autorizzazione è concessa per l'area che è compresa nel rettangolo che ha per vertici le coordinate 45.45°N, 12.25°E e 45.30°N, 12.45°E.

Following the attached request, acquired at the protocol of the writer General Directorate with protocol n. 19970/STA on 22 September 2017, under the terms of the CC-BY4.0 "Creative Commons Attribution License" (CCAL), the publication of the images used in the article "Fast shoreline erosion induced by ship wakes in a coastal lagoon: field evidence and remote sensing analysis "derived from the WMS Service named "Ortofoto a colori anno 2012 con relative date del volo", published by the Italian National Geoportal (http://www.pcn.minambiente.it/mattm/), is authorized.

The permission is granted for the area comprised within the rectangle individuated by the coordinates 45.45°N, 12.25°E e 45.30°N, 12.45°E

IL DIRETTORE GENERALE

Dott.ssa Gaia Checcucci
